# Posterior Reversible Encephalopathy Syndrome in a Patient with Post Streptococcal Glomerulonephritis: A Case Report

**DOI:** 10.31729/jnma.5065

**Published:** 2020-12-31

**Authors:** Deependra Mandal, Deepa Khanal, Rajan Phuyal, Anwesh Bhatta

**Affiliations:** 1 Kathmandu Medical College and Teaching Hospital, Sinamangal, Kathmandu, Nepal; 2 Department of Pediatrics, Kathmandu Medical College and Teaching Hospital, Sinamangal, Kathmandu, Nepal

**Keywords:** *hypertensive emergency*, *reversible encephalopathy*, *tonic-clonic seizure*, *vision loss*

## Abstract

Posterior reversible encephalopathy syndrome is a clinical-radiological syndrome neurological disorder with varied symptoms which includes headache, visual field defects, seizures, altered consciousness. It is a rare complication of post-streptococcal glomerulonephritis and results in life-threatening manifestation if not managed on time. Although reversible by definition, complications like status epilepticus, intracranial hemorrhage, and ischemic infarction may lead to mortality and morbidity. We report a case of a 9-year-old female patient with posterior reversible encephalopathy syndrome who presented with multiple episodes of seizures and bilateral painless loss of vision for 1 day. Due to her severity, a computed tomography scan was sent which revealed a hypodense lesion in the brain. She was admitted to the pediatric intensive care unit and managed with supportive care for 6 days where she died on the 6th day. Vital signs are simple but important and if overlooked can lead to a series of complicated events.

## INTRODUCTION

Posterior reversible encephalopathy syndrome (PRES) is a neurological disorder characterized by neurological signs and symptoms with vasogenic edema.^1^ The hypothesis proposes a rapid increase of arterial blood pressure up to a hypertensive crisis or emergency, in a majority of patients with PRES.^1^ Complication of PRES includes status epilepticus, intracerebral hemorrhage, intracranial hypertension, or cerebral ischemia. PRES has been reported in almost all age groups, but most frequently in young or middle-aged adults with female preponderance.^2,3^ We report an unusual case of a 9-year female with post-streptococcal glomerulonephritis. She had complications of posterior reversible encephalopathy syndrome and hypertensive emergency secondary to post-streptococcal glomerulonephritis.

## CASE REPORT

A 9-year-old girl from Khotang presented to the emergency department with a history of acute onset of headache for 3 days, altered sensorium, multiple episodes of tonic-clonic seizures lasting each for 2-3 minutes, and sudden bilateral painless loss of vision for 1 day. She initially visited a nearby district hospital from where she was referred to a higher center for ophthalmologic evaluation. Due to her altered sensorium, she was first referred to our center for pediatric evaluation before eye problems could be addressed.

On examination, she was afebrile, irritable with altered sensorium GCS 11/15 (E4V2M5), mild puffiness of the face, and bilateral pitting edema. Her blood pressure (BP) was 150/100 mm Hg (>99th percentile for her age and height), SPO2 was 78% on room air. Her other vital parameters and anthropometry were normal. Her pupils were bilaterally equal and reacting to light, with normal fundus examination. She had no focal neurological deficits except for generalized exaggerated deep tendon reflexes and had no signs of any meningeal irritation. Other systemic examinations were normal. She was managed at the Pediatric Intensive Care Unit (PICU) with oxygen at facemask on 4L/min, Inj. Mannitol, Inj. Furosemide, Tab Enalapril, Inj. Ceftriaxone and Inj. Phenytoin and other supportive care. Her BP was carefully monitored.

**Figure 1:**
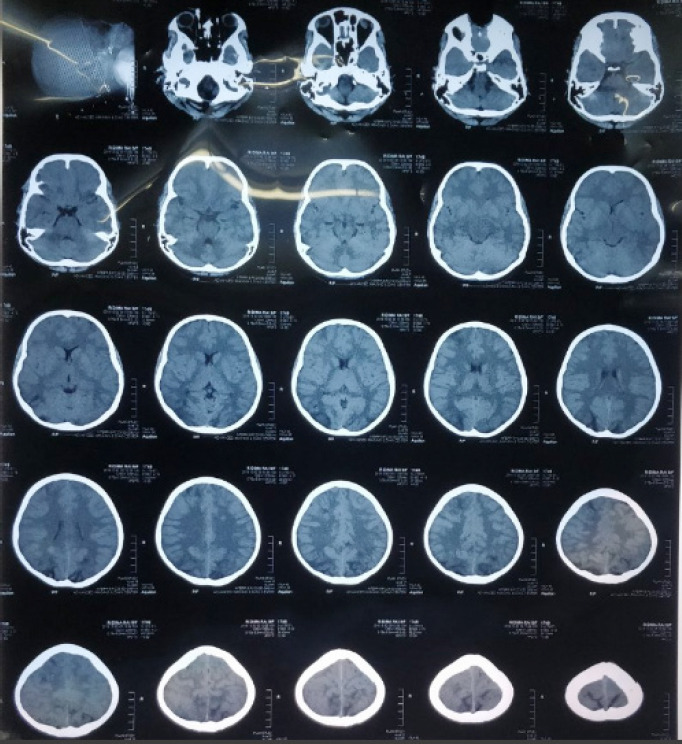
CT of the brain showing an ill-defined hypodense lesion in the bilateral occipital region.

Investigations revealed hemoglobin 11.4gm/dl, total count 21,000 cells/mm^3^ (polymorphs 85%, lymphocytes 13%), platelets 4,60,000 cells/mm^3^; C-reactive protein negative; raised blood urea 58mg/dl; and creatinine 0.5 mg/dl. Her serum electrolytes and liver function tests were normal. Urinalysis revealed plenty of RBCs with albumin trace and pus cell 2-3 /HPF. The chest X-ray was normal and the ultrasonogram of the abdomen revealed mild ascites. ECG showed sinus tachycardia. Computed tomography (CT) of the brain revealed an ill-defined hypodense lesion in the bilateral occipital region, adjacent parietal lobes, and frontal lobe mainly involving the white matter, s/o white matter edema (Figure 1).

Anti-streptolysin-O (ASO) titer was positive (>800 Units/ml) and complement C^3^ level was low (8.5 mg/ dl). Blood and throat swab cultures were sterile.

Her blood pressure was persistently rising>99^th^ percentile even after initiation of Enalapril, Mannitol, and Furosemide. Inj. Labetalol was started on continuous infusion, following which her blood pressure gradually subsided and her vision returned to normal on 2^nd^ day of admission and could recognize her father. Her blood pressure reached to 95^th^ percentile and edema started to decrease.

A diagnosis of hypertensive emergency secondary to acute post-streptococcal glomerulonephritis was made which is also called posterior reversible leukoencephalopathy syndrome.

**Figure 2:**
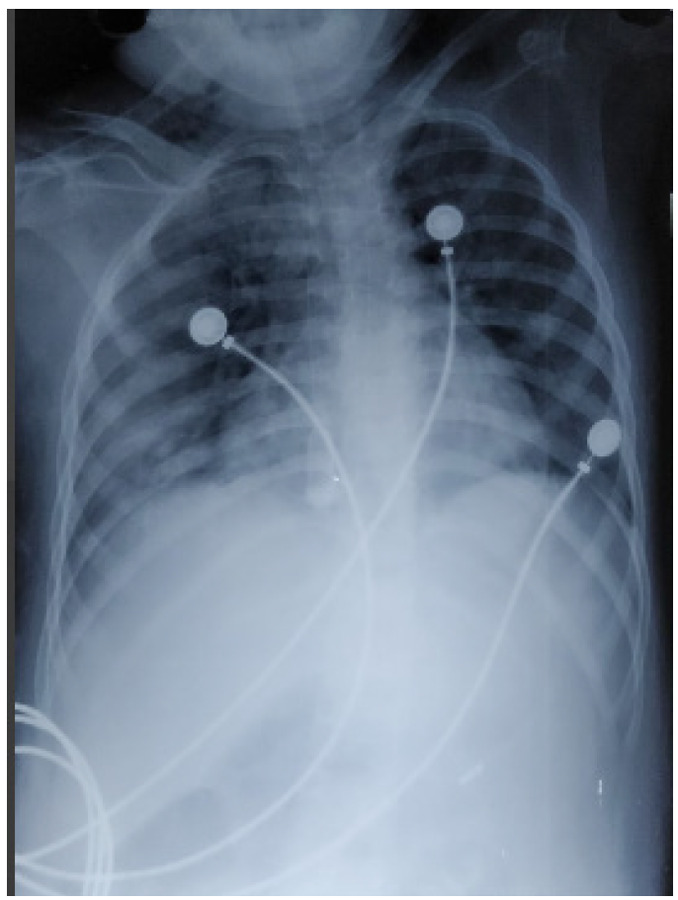
Chest X-ray showing pulmonary edema.

On 3^rd^ day of the PICU stay, she developed another complication of glomerulonephritis in the form of pulmonary edema which warranted mechanical ventilation. A chest X-ray showed pulmonary edema (Figure 2).

She was managed for pulmonary edema with Furosemide, Dobutamine, and continuing mechanical ventilation. However, on the 6th day of admission, she was declared dead secondary to refractory pulmonary edema.

## DISCUSSION

Posterior reversible encephalopathy syndrome (PRES) was first described in 1996.^4^ Developing posterior reversible encephalopathy syndrome following post-streptococcal glomerulonephritis is very rare.^5^ There are many hypotheses but no literature has proven the underlying pathophysiology of PRES. Among many hypotheses, the theory explaining the increase in blood pressure to a hypertensive emergency leading breakdown in cerebral autoregulation is widely accepted. The combination of hypertension along with endothelial damage leads to hydrostatic edema giving rise to vasogenic edema. When this edema is severe enough, radiographic evidence can be found.

The majority of patients with PRES present with headaches, loss of vision, and seizures.^6,7^ These symptoms were present in our case as well. The clinical presentation includes elevated arterial blood pressure up to hypertensive emergencies.^1^ Evaluation of our patient for the underlying cause of hypertension revealed investigation finding supportive of acute nephritic syndrome secondary to Streptococcal infection. Almost similar findings were reported earlier.^8^

The radiologic abnormalities in PRES are evident in CT scan as white matter edema typically most prominent in both posterior cerebral hemispheres. Our case had similar neuroimaging findings as reported by Prasad et al.^7^ The diagnosis was based on the presence of a hypertensive emergency, typical CT finding, and suggestive investigation. Although MRI is the gold standard^4^ and should be performed as soon as PRES is suspected, a CT scan was evident enough to make a diagnosis in this case. If promptly recognized and treated with appropriate drugs for hypertension and cerebral edema, the clinical syndrome usually resolves within a week.^2,3^

The pediatric population is more vulnerable to cerebrovascular dysfunction than adults with systemic involving diseases like hypertension. PRES can present with an unusual symptom like loss of vision and can often be missed if simple vital signs are overlooked. In a patient with loss of vision with hypertension, PRES should be kept as a differential and further workup needs to be done.
